# Improvement of Model Predictive Current Control Sensing Strategy for a Developed Small Flux-Switching Permanent Magnet Motor

**DOI:** 10.3390/s20113177

**Published:** 2020-06-03

**Authors:** Cheng-Tang Pan, Shao-Yu Wang, Chun-Chieh Chang, Chung-Kun Yen, Jyun-Yi Wu, Shin-Pon Ju, Roger Cheng-Lung Lee

**Affiliations:** 1 Department of Mechanical and Electro-Mechanical Engineering, National Sun Yat-sen University, Kaohsiung 80424, Taiwan; pan@mem.nsysu.edu.tw (C.-T.P.); sywang@mem.nsysu.edu.tw (S.-Y.W.); a0988852720@gmail.com (C.-C.C.); alden0113@gmail.com (C.-K.Y.); zx71047788@gmail.com (J.-Y.W.); 2 Institute of Medical Science and Technology, National Sun Yat-sen University, Kaohsiung 80424, Taiwan; 3 Office of Research and Development, Naroller Electronics Co., Ltd., Taoyuan City 33393, Taiwan

**Keywords:** model predictive current control (MPCC), flux-switching permanent magnet motor (FSPM), uniform design method, cogging torque, torque sensing

## Abstract

This paper presents an improved control system for a small flux-switching permanent magnet motor (FSPM) to enhance its performance and torque sensing. The analytical magnetic circuit design was used to determine the related motor parameters, such as the air gap flux density, permeance coefficient (Pc), torque, winding turns, pole number, width, length, magnet geometry, and the current density of FSPM. The electromagnetic analysis of this motor was performed by software (ANSYS Maxwell) to optimize the motor performance. In this study, the performance of FSPM was investigated by the uniform design experimentation (UDE). For the control system, the model predictive current control (MPCC) is currently recognized as a high-performance control strategy, due to its quick response and simple principle. This model contained the nonlinear part of the system, to improve the torque ripple of FSPM. A modified MPCC strategy was proposed to improve the distortion of the current waveform and decrease the computational burden. The new modified control architecture was mainly composed of three parts, such as the estimation of electromotive force (EMF), current prediction, and optimal vector selection/vector duration. When the reference voltage vector was obtained, the three-phase duties were easily determined by the principle of space vector modulation (SVM). The results show the different strategy methods between the newly proposed modified MPCC and traditional proportional integral (PI) controller. In the control of FSPM, a modified MPCC strategy was able to achieve a better performance response and decrease the computational burden. At a low speed of 350 rpm, the proposed modified MPCC can achieve a better dynamic response. The nonlinear problem of the startup speed was also effectively resolved. The torque sensing performance of the simulation and the experimental test value were compared. The torque sensing performance of the simulation and the actual test value were also examined. In this study, the optimization focused not only on the motor design and fabrication, but also on an improved motor control strategy and torque sensing, in order to achieve the integrity of the FSPM system.

## 1. Introduction

With increasing demand for high-performance motors in various applications from aerospace and automotive to medical equipment, flux-switching permanent magnet (FSPM) motors have aroused considerable attention, due to their high-power densities and high efficiencies. The idea of the FSPM motor was proposed in 1955 [1]. For good combination of rotor-inserted permanent magnet machine and switched reluctance machine, the FSPM motor has demonstrated attractive merits of high torque density, strong mechanical robustness, good thermal dissipation ability, etc. However, the FSPM motor with inherited double salient structure suffers from severe torque ripple and speed ripples, as well as acoustic noise and vibration, particularly in high-power or low-speed applications, because of its serious nonlinear relationship between flux linkage and phase current [2,3]. To reduce the torque ripple, lots of research works have been done since the 1990s [4,5,6,7], most of which are focused on optimal electromagnetic design. To solve the above problems, various control methods have been proposed in the literature.

One control method was used by modifying the injected stator excitation currents and producing an additional torque component to counteract the torque ripple [8]. Jia et al. [9] investigated a harmonic spectrum analysis on the cogging torque. Then, by injecting harmonic current into a 12/10 pole FSPM motor, the influence of cogging torque was reduced. Other current control methods may include repetitive current control and high-bandwidth current control techniques, etc. [10,11]. However, this method only considers the main high-order harmonic, while ignoring the rest of the high-order harmonics. Stator armature currents are also considered specifically to produce large total harmonic distortion (THD). In addition, Zhu et al. [12] considered the periodic characteristics of the cogging torque of the FSPM motor, and a new control method was proposed to compensate for the cogging torque of FSPM motor. The advantages of iterative learning control (ILC) and direct torque control (DTC) based on space vector modulation (SVM) were combined in the control system. However, the steady-state torque ripple was still high, especially for permanent magnet brushless motors with large cogging torques. A neural network predictive controller (NPCC) [13] was proposed to predict the estimates of variables, such as generator speed or blade pitch angle. This required considerable training time and many learning layers to predict accurately. Recently, model predictive control (MPC) has emerged as a powerful scheme for high-performance control of PMSM drives [14,15,16,17,18] as well as power converters [19,20,21,22]. Based on the internal model of the system, MPC predicts the future behavior of controlled variables, such as current, torque, and stator flux. By minimizing the error between the reference value and the predicted value, the best voltage vector can be obtained. It eliminates the current regulators and pulse width modulation (PWM) block in vector control (VC), and selects the best voltage vector by minimizing a cost function. Furthermore, MPC easily handles multi-variable control, and considers various nonlinear constraints, featuring high flexibility [23,24]. Among them, model predictive current control (MPCC) for permanent magnet synchronous machine (PMSM) drives has been widely recognized as a high-performance control strategy with quick response and simple principle [25]. In 2014, the MPCC model was first applied to the FSPM motor [26], which effectively reduced the torque ripple, but also caused the distortion of the current waveform.

In spite of the advantages of MPC, it also has some drawbacks. As only one voltage vector is applied during one control period, it produces relatively high steady-state ripples, and the current harmonics are distributed over a wide frequency range [27]. Furthermore, the cost function is evaluated for each converter voltage vector, which is computationally intensive and poses a high computational burden on the hardware [25,28]. To cope with the problems of conventional MPC, various improved MPC methods have been proposed in the literature. The steady-state performance of conventional MPC can be improved by applying one non-zero vector and a zero vector during one control period [25,28,29], where the duration of the non-zero vector is determined based on certain principles, such as current error minimization [28]. Therefore, the acquisition of the approximately linearized transfer function of the controller design for the FSPM motor becomes complicated, and also leads to robustness problems. For today’s VC control, the inner loop proportional integral (PI) controller can only handle linear systems, so there are many restrictions on control [23]. The MPC-based model can contain the nonlinear part of the system and calculate the appropriate current value. On the other hand, the MPC model can predict the future behavior of controlled variables [25,30], such as current, torque, and stator flux. Therefore, MPC is more suitable for applications than VC, where very high dynamic response is required. This is also a major feature of MPC, but still need to improve it. The current MPC control architecture selected the optimal voltage vector with a minimum cost function. The cost function was sampled from eight sets of data, which is really time-consuming, and the control accuracy and the fast response were lacking. In addition, it produced relatively high steady-state ripple, and current harmonics were distributed over a wide frequency range [27]. These results show limits in applications, such as robots, machine tools, medical equipment, etc. Therefore, this paper proposes a modified MPCC architecture based on the MPC architecture. The traditional cost function is replaced by the drive method of SVM, to reduce the computational complexity and achieve higher control accuracy and current harmonics. Also, the proposed prediction voltage model considers the components of the inductor, which can effectively reduce the torque ripple caused by the reluctance torque of the FSPM motor. With the current prediction model, the response time of the motor can be improved.

The design principle of this permanent magnet motor was applied to the FSPM motor to illustrate an integrated design rule of FSPM and verify its feasibility. FSPM with high power density and easy-to-dissipate thermal heat during operation is a relatively new topology of a brushless motor. The complete FSPM motor design flow and the motor control method were proposed. In the structure design portion of this paper, the sizes were substituted into the analytical mathematical model for rough derivation. The parameters were imported into the simulation software analysis, and finally optimized by the uniform experiment method (UDE), through completing a series of design processes. This paper also proposes a modified MPCC strategy to achieve a better dynamic performance and lower torque ripple. In addition, the modified MPCC strategy was able to decrease the computational burden and solve the nonlinear problem of the startup speed. The new control architecture was mainly composed of three parts, which are the estimation of electromotive force (EMF), current prediction, and optimal vector selection/vector duration. Based on the principle of SVM, when the reference voltage vector was obtained, the three-phase duties can be obtained.

## 2. Research Methods

### 2.1. The Specifications and Dimensions of FSPM

**Figure 1:**
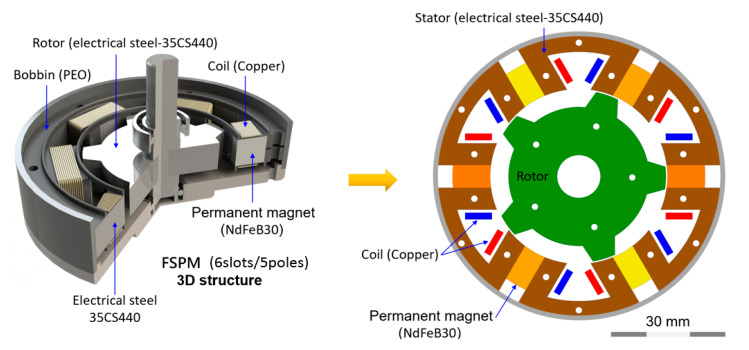
The schematic diagram of 6-slots/5-poles (6/5) flux-switching permanent magnet (FSPM).

The rated specifications of FSPM were defined, and the parameters were determined carefully for industrial application purpose. In this study, six stator slots with five rotor poles of FSPM was investigated, which was called 6-slots/5-poles FSPM (6/5 FSPM) in the following section. The 6/5 FSPM structure is schematically shown in Figure 1. Specifications of 6/5 FSPM are shown in Table 1.

**Figure 2:**
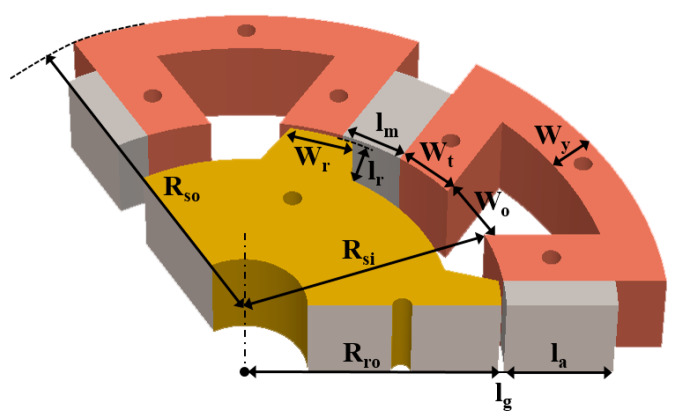
The symbolic description of the as-designed 6/5 FSPM.

After the FSPM specifications were decided, the detailed stator dimensions were calculated, whose symbolic description and dimension are shown in Figure 2. The inner stator radius (*R_si_*) was obtained by Equation (1). The stator tooth width (*w_t_*), the stator yoke width (*w_y_*), the stator slot opening (*w_o_*), the magnet width (*l_m_*), and the rotor salient width (*w_r_*) were set as the same dimension, and evenly distributed to the inner stator circumference, as shown in Equation (2), where N_s_ is slot number.

(1) $$R_{si}=K_{sp}\times R_{so}$$

(2) $$w_{t}=w_{y}=w_{o}=l_{m}=\frac{2\pi \times R_{si}}{4\times N_{s}}$$

### 2.2. The Winding Configuration of FSPM

In the winding configuration design of the 6/5 FSPM, the feasibility of the three-phase winding was verified first. The *K* value in Equation (3) was a positive integer, to confirm the combination of slot number and pole number was a suitable choice.

(3) $$K=\frac{N_{s}}{m\cdot GCD(N_{s},N_{p})}=\frac{6}{3\times GCD(6,5)}=2$$

where *N_p_* is pole number, and GCD is the highest common factor.

The angle of the *k*th slot was obtained by Equation (4) and introduced into the phase offset expression, as shown in Equation (5).

(4) $$\theta_{s}(k)=k\frac{N_{p}}{N_{s}}360^{\circ },\;\mathrm{for}\;k=1,2,\ldots ,N_{s}-1\;$$

(5) $$K_{0}=\frac{120^{\circ }+q\times 360^{\circ }}{\theta_{s}}=\frac{N_{s}}{3N_{p}}(1+3q)=\frac{6}{3\times 5}(1+3q)$$

where *q* is any integer. In the case of the 6/5 FSPM, when *q* was equal to 2, the valid phase offset $K_{0}$ was obtained as 4.

Finally, the coil span slot number S* was calculated as 1 by Equation (6). The meaning of S* = 1 is that the coil of 6/5 FSPM was a concentrated winding configuration.

(6) $$S^{*}=\max\left[fix\left(\frac{360}{\theta_{s}}\right),1\right]=\max\left[fix\left(\frac{6}{5}\right),1\right]=\max\left[1,1\right]=1$$

After confirming the concentrated winding method, the winding configuration was established and is shown in Table 2. The electrical angles were sequentially increased by, and according to, the winding slot number. The in and out slots of the even-numbered slot coils were reversed to eliminate the 180° electrical angle caused by the permanent magnet. Then, 360° simplifications and 180° simplifications were executed respectively. Finally, the two slots closest to 0° were selected to form a complete A-phase winding. The A-phase winding number was added with *K*_0_ and 2*K*_0_ to obtain the slots numbers of B-phase and C-phase, respectively.

### 2.3. The Magnetic Analysis and Winding Turns of FSPM

The equivalent magnetic circuit analysis was performed to calculate the output performance of the motor after deciding the input FSPM parameters, which were used to derive the math model regarding the flux density of the air gap and the permanent magnet. This method is based on the magnetic field which is similar to the electric field, such as the voltage corresponding to the magnetomotive force, and the current corresponding to the magnetic flux density. In this paper, the magnetic flux density and FSPM output performance were analyzed preliminarily by ANSYS Maxwell software.

**Figure 3:**
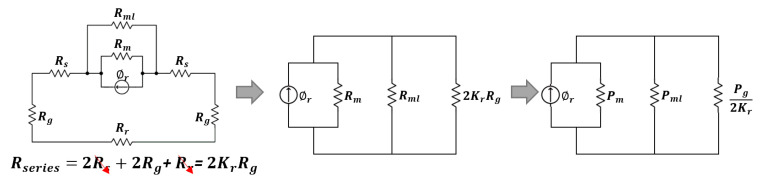
The magnetic circuit of FSPM.

Figure 3 shows the flux route of FSPM, where $R_{r}$ is the rotor magnetic reluctance, $R_{s}$ is the stator magnetic reluctance, $R_{g}$ is the air gap magnetic reluctance, $R_{m}$ is the magnet magnetic reluctance, and $R_{ml}$ is the leakage magnetic reluctance. Since $R_{s}$ and $R_{r}$ are far less than the $R_{g}$, and for calculation convenience, these two reluctances were omitted, to simplify the flux route.

Equation (7) shows the definition of permeance (P), which is the inverse of magnetic reluctance. The flux route was transferred into Equation (8), based on Ohm’s Law and the Kirchhoff Circuit Laws.

(7) $$P=\frac{\mu A}{l}$$

(8) $$B_{g}=\frac{P_{g}}{P_{g}+K_{r}(2P_{m}+2P_{ml})}B_{m}\frac{A_{g}}{A_{m}}$$

where $A_{m}$ is the magnet area, $A_{g}$ is the air gap area, μ is permeability, $B_{g}$ is the air gap flux density, $P_{m}$ is permanent magnet permeance, $P_{ml}$ is leakage magnet permeance, and $K_{r}$ is the compensating coefficient (1.05–1.55). Since $P_{ml}$ is far less than $P_{m}$, it was omitted for calculation convenience.

**Figure 4:**
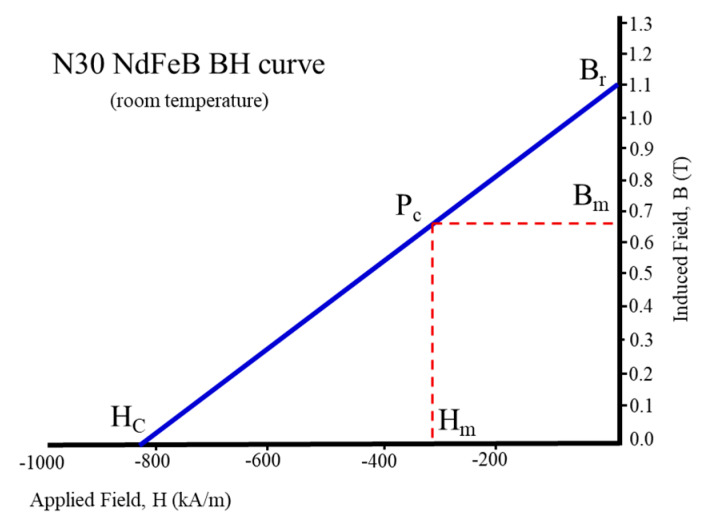
The hysteresis curve of the N30 NdFeB permanent magnet.

The magnet flux density $B_{m}$ was derived from the magnetic field $H_{m}$ based on the BH curve, as shown in Figure 4. The definition of permeance coefficient $P_{c}$ is shown by Equation (9). The magnet field strength $H_{m}$ was derived in Equation (10) by the Rowland’s Law and the definition of $P_{c}$, where $\mu_{0}$ is vacuum permeability, $\mu_{r}$ is relative permeability, and $B_{r}$ is remanence.

(9) $$P_{c}=\frac{B_{m}}{H_{m}}=\mu_{0}\frac{l_{m}A_{g}}{l_{g}A_{m}}$$

(10) $$H_{m}=\frac{B_{r}}{\mu_{0}\mu_{r}+P_{c}}$$

The magnetic flux analysis needed to consider the winding turns and determine whether the design was within a reasonable range. Since the copper windings will directly affect the voltage waveform, coil self-inductance, and torque ripple of FSPM, it had to be calculated appropriately. Based on Equation (11), the total winding turns N were derived from the torque equation:

(11) $$T=k_{w}\times N\times B_{g}\times \frac{N_{r}}{\pi }\times A_{g}\times I$$

where $k_{w}$ is the winding factor, $N_{r}$ is the pole number, and I is the input current.

### 2.4. Proposed Modified Model Predictive Current Control

**Figure 5:**
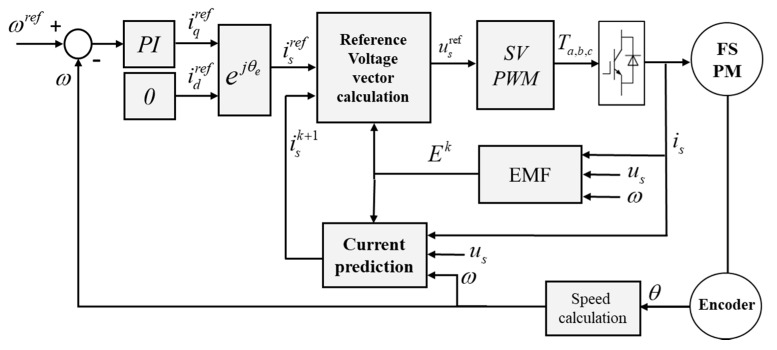
The as-proposed modified model predictive current control (MPCC) control strategy.

**Figure 6:**
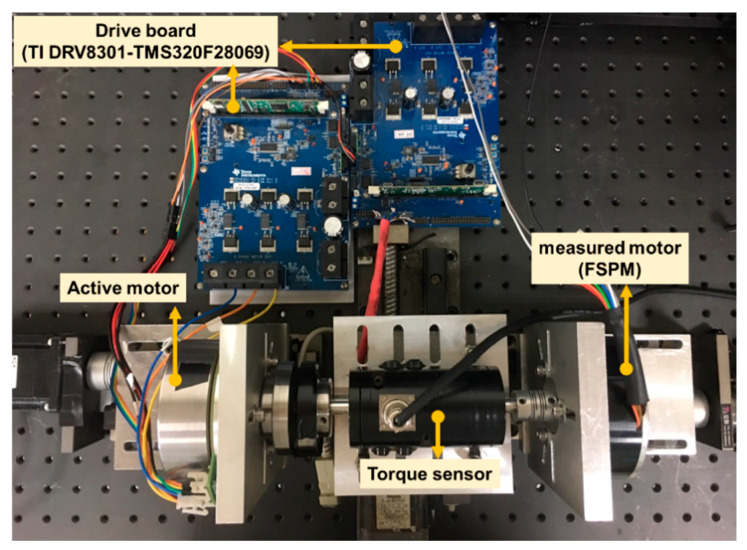
Schematic diagram of experimental setup.

The control diagram of the proposed modified MPCC had the performance of fast response and simple principle. This model also had a nonlinear portion that can improve torque ripple, due to the doubly salient effect of the FSPM rotor structure [3]. It consisted of back electromotive force (back-EMF) estimation, current prediction, and voltage vector, as shown in Figure 5. The real-time control MCU (TI DRV8301-TMS320F28069) for algorithm implementation was used to achieve the control strategy, and 10 kHz was selected as the sampling frequency. The signals of the current and speed were output by the D/A converter. The experimental setup is shown in Figure 6. Compared with the traditional MPCC [26], the part of the cost function was replaced by the drive method of SVM, and the voltage model was proposed to obtain the voltage vector in this paper. When the final voltage vector output was obtained, the three-phase duty ratio could be easily calculated by SVM. Then, a six-bridge switch with three sets of PWM switches controlled three-phase current to the motor. In the feedback system, an incremental encoder was used to obtain the angle signal and calculate the angular velocity for the back-EMF estimation, current prediction, and speed PI controller.

Electrical torque was expressed as Equation (12), including reluctance torque (*T_rs_*), cogging torque (*T_cog_*), and magnet torque (*T_pm_*). *T_pm_* is the PM torque produced by the interaction between the armature winding current and PM magnetic field, *T_rs_* is the reluctance torque due to the magnetic reluctance variations with rotor positions, and *T_cog_* is the cogging torque generated by PM field energy alterations with rotor positions.

(12) $$T_{es}=\frac{1}{2}i_{s}^{2}\frac{\partial L_{s}}{\partial \theta_{\gamma }}+i_{s}\frac{\partial \psi_{pm}}{\partial \theta_{\gamma }}-\frac{\partial W_{pm}}{\partial \theta_{\gamma }}=T_{rs}+T_{pm}+T_{cog}$$

The definition of torque ripple *T_ripple_* can be given by:

(13) $$T_{ripple}=\frac{T_{es\_\max}-T_{es\_\min}}{T_{es\_avg}}$$

where *T_es_max_*, *T_es_min_*, and *T_es_avg_* are the maximum, minimum, and average values of electromagnetic torque.

The torque ripple could be improved by considering the effects of mutual inductance in the control. However, in many literatures [25,27,28], the mutual inductance of FSPM was ignored, which may bring some defects to the accuracy of the model. Therefore, this paper considers the formula of the inductor [26] in the control architecture. To simplify the theoretical analysis, some assumptions are given as follows: (1) iron saturation is negligible; and (2) eddy current and core loss have not been considered. Based on the assumptions mentioned above, the PM flux, inductance, and mutual inductance are supposed ideally sinusoidal. The mathematical equations of three-phase inductance can be expressed as:

(14) $$\left\{\begin{array}{l} L_{A}=L_{o}-L_{i}\cos(2\theta_{\gamma })\\ L_{B}=L_{o}-L_{i}\cos(2\theta_{\gamma }+2\pi /3)\\ L_{C}=L_{o}-L_{i}\cos(2\theta_{\gamma }-2\pi /3) \end{array}\right\}$$

where *L_o_* and *L_i_* are the inductance initial value and amplitude, respectively.

In this paper, the mutual inductance of FSPMSM is considered as:

(15) $$\left\{\begin{array}{l} L_{AB}=L_{BA}=-\frac{1}{2}L_{o}+L_{i}\cos2(\theta_{\gamma }-\frac{2\pi }{3})\\ L_{BC}=L_{BC}=-\frac{1}{2}L_{o}+L_{i}\cos2(\theta_{\gamma }+\frac{2\pi }{3})\\ L_{AC}=L_{CA}=-\frac{1}{2}L_{o}+L_{i}\cos2(\theta_{\gamma }+\pi ) \end{array}\right\}$$

(16) $$L_{sp}=\left\{\begin{array}{ccc} L_{A} & L_{AB} & L_{AC}\\ L_{BA} & L_{B} & L_{BC}\\ L_{AC} & L_{BC} & L_{C} \end{array}\right\}$$

In the program operation, to effectively resolve the nonlinear factors and reduce the complexity of the calculation, the model of the FSPM was represented by the stationary coordinate system α-β. The total inductance of the motor winding (Equation (16)) was followed by using Clarke transformation and input to the model of FSPM. The model of FSPM could be derived by the coordinates of the complex vector, which were expressed as:

(17) $$u_{s}=R_{s}i_{s}+\frac{d}{dt}(L_{s}i_{s}+\psi_{pm})=R_{s}i_{s}+L_{s}\frac{di_{s}}{dt}+2L_{s}i_{s}\sin(2\theta_{\gamma })\omega_{\gamma }+\frac{d\psi_{pm}}{d\theta_{\gamma }}\omega_{\gamma }$$

where $\psi_{pm}=\psi_{f}e^{j\theta_{e}}$ is the rotor flux vector; *R_s_*, *L_s_*, *u_s_*, *i_s_*, *ω_γ_*, and *θ_γ_* are the stator resistance, inductance, stator voltage phasor, stator current phasor, rotor speed, and rotor angle, respectively.

**A. Back Electromotive Force (Back-EMF) Estimation [28]**

Since the back-EMF could not be directly measured, it was necessary to estimate, using the voltage equation. Moreover, since the electrical time constant was more than the mechanical time constant, and the back-EMF was linear with the rotor speed (*ω_γ_*), it could be assumed that the back-EMF was constant for several consecutive control periods. By letting *E^k^* = $\frac{d\psi_{pm}}{d\theta_{\gamma }}\omega_{\gamma }$ in Equation (17), the back-EMF equation could be obtained as:

(18) $$E^{k-1}=u_{s}^{k-1}-R_{s}\frac{i_{s}^{k}+i_{s}^{k-1}}{2}-\frac{L_{s}}{T_{sc}}(i_{s}^{k}-i_{s}^{k-1})-2L_{s}i_{s}^{k-1}\sin(2\theta_{\gamma }^{k-1})\omega_{\gamma }$$

where *T_sc_* is the sampling period and $i_{s}^{k}$ is the measured phase current. Similarly, the *E ^k−2^* and *E ^k−3^* could be obtained as:

(19) $$E^{k-2}=u_{s}^{k-2}-R_{s}\frac{i_{s}^{k-1}+i_{s}^{k-2}}{2}-\frac{L_{s}}{T_{sc}}(i_{s}^{k-1}-i_{s}^{k-2})-2L_{s}i_{s}^{k-2}\sin(2\theta_{\gamma }^{k-2})\omega_{\gamma }$$

(20) $$E^{k-3}=u_{s}^{k-3}-R_{s}\frac{i_{s}^{k-2}+i_{s}^{k-3}}{2}-\frac{L_{s}}{T_{sc}}(i_{s}^{k-2}-i_{s}^{k-3})-2L_{s}i_{s}^{k-3}\sin(2\theta_{\gamma }^{k-3}+\alpha )\omega_{\gamma }$$

Then, to avoid errors caused by the operation process, *E^k^* could be used as the average of back-EMF in the past several control cycles, as shown in Equation (21). A detailed analysis of the robustness of the back-EMF estimation was discussed. The average of E was used twice, three times, and five times in the past. An error percentage was used to compare its error percentage, as shown in Equation (22). From the previous tests, the error percentages of the average of 2, 3, and 4 times were found to be 92%, 98%, and 99%, respectively. Therefore, the average of back-EMF was determined to be used three times in the past as the formula.

(21) $$E^{k}=\sum_{k=1}^{N}E(k)/N$$

(22) $$e=\frac{E^{k’}-E^{k}}{E^{k}}\times 100\%$$

where *e* and *E^k^*^’^ are the error percentage and the previous step of back-EMF.

Finally, *E^k^* could be used as the average of back-EMF in the past three control cycles. In addition, one of the main advantages was that it avoids the precise machine parameters, rotor speed and rotor position [31].

**B. Current Prediction**

There were various delays that caused an insufficient response in the controller, including sampling delays, filtering delays, and other factors. In the digital control implementation, the value of the command current vector at present could not be applied immediately, so the prediction of the transmitted current could obtain the value of the *k* + 1 to achieve the best response. In the paper, compared to the traditional MPCC strategy [26], the proposed current prediction was obtained in the stationary coordinate system α-β, and the torque ripple was effectively improved. By adjusting Equation (17), the current variation could be calculated as:

(23) $$\frac{di_{s}}{dt}=\frac{\left(u_{s}-R_{s}i_{s}-2L_{s}i_{s}\sin(2\theta_{\gamma })\omega_{\gamma }-E_{s}\right)}{L_{s}}$$

Then, the next predicted current was obtained as:

(24) $$i_{s}^{k+1}=i_{s}^{k}+\frac{1}{L_{s}}(u_{s}-R_{s}i_{s}^{k}-2L_{s}i_{s}\sin(2\theta_{\gamma })\omega_{\gamma }-E_{s}^{k})T_{s}$$

**C. Voltage Vector Prediction**

According to Equation (17), the voltage vector could be obtained during *k* and *k* + 1:

(25) $$u_{s}^{k}=R_{s}i_{s}+\frac{L_{s}}{T_{sc}}(i_{s}^{k}-i_{s}^{k-1})+2L_{s}i_{s}\sin(2\theta_{\gamma })\omega_{\gamma }+E^{k}$$

In the paper, the calculations of the reference current $i_{p}^{ref}$ by the PI controller and the predicted stator current $i_{p}^{k+1}$ using Equation (24) were introduced to Equation (25). Then, the reference voltage vector was obtained using Equation (26) and could increase the response speed of the motor.

(26) $$u_{p}^{ref}=R_{p}\frac{i_{p}^{ref}+i_{p}^{k+1}}{2}+\frac{L_{s}}{T_{sc}}(i_{p}^{k+1}-i_{p}^{k})+2L_{s}i_{s}\sin(2\theta_{\gamma })\omega_{\gamma }+E^{k}$$

After obtaining the reference voltage vector, the control of the three-phase voltage was achieved by the driving mode of SVM. For the traditional MPCC, it used a non-zero voltage vector with a zero vector during the control period [28]. Although the control switching frequency could be reduced, the control accuracy was insufficient, and the nonlinear FSPM could not be satisfied. In the proposed control strategy, the nonlinear prediction model was combined with the SVM driving method to reduce the torque ripple and further improve the response.

## 3. Results and Discussion

### 3.1. The Calculation Results of the Magnetic Flux Analysis and Simulation

The outer stator radius (*R_so_*) was limited to 43 mm, and the stator split ratio (*K_sp_*) was usually set between 0.5 and 0.65. The stator tooth width (*w_t_*), the stator yoke width (*w_y_*), the stator slot opening (*w_o_*), the magnet width (*l_m_*), and the rotor salient width (*w_r_*) were all the same dimension in 6.9 mm; the initial dimensions of 6/5 FSPM are shown in Table 3. Table 4 shows the calculation results of the magnetic flux analysis. The lengths of magnet and airgap were defined as 6.9 mm and 0.5 mm, respectively. The cross-sectional area is the magnetic flux passing area. The flux densities of magnet (*B_m_*) and air gap (*B_g_*) were calculated as 0.88 T and 0.85 T by Equation (8) to Equation (10). The winding turns per phase (*N_ph_*) was calculated based on the rated speed of 1000 rpm and other required specifications.

**Figure 7:**
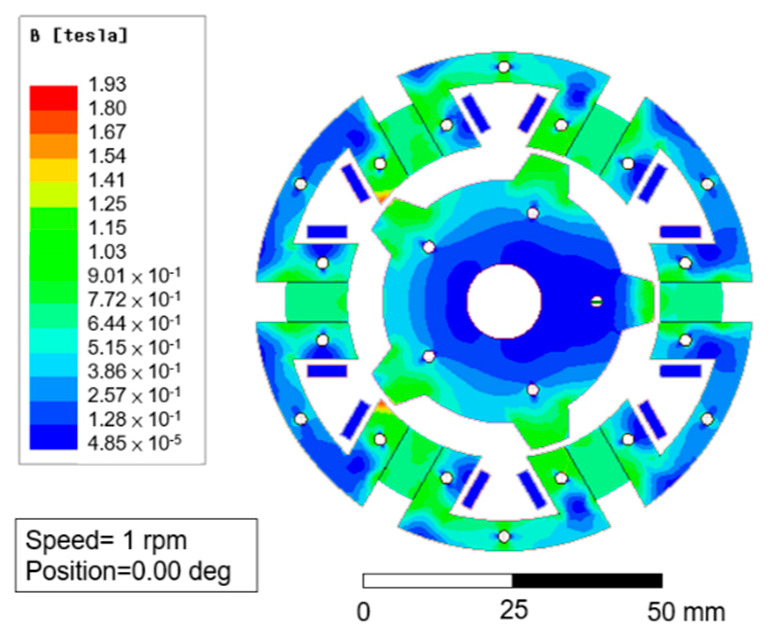
The magnetic flux density of 6/5 FSPM structure.

To optimize the parameters, these parameters were all analyzed by ANSYS Maxwell and shown as follows. Figure 7 shows the magnetic flux density of the FSPM structure. The magnetic saturation occurred when the stator teeth were directly facing the rotor teeth. The phenomenon caused heat concentration and reduced efficiency. This problem was resolved by modifying the structure, such as shortening the magnet length. The simulation result of B_g_ was about 0.8–1.0 T, which was similar to the calculation result 0.85 T.

### 3.2. The Cogging Torque Optimization of FSPM

**Figure 8:**
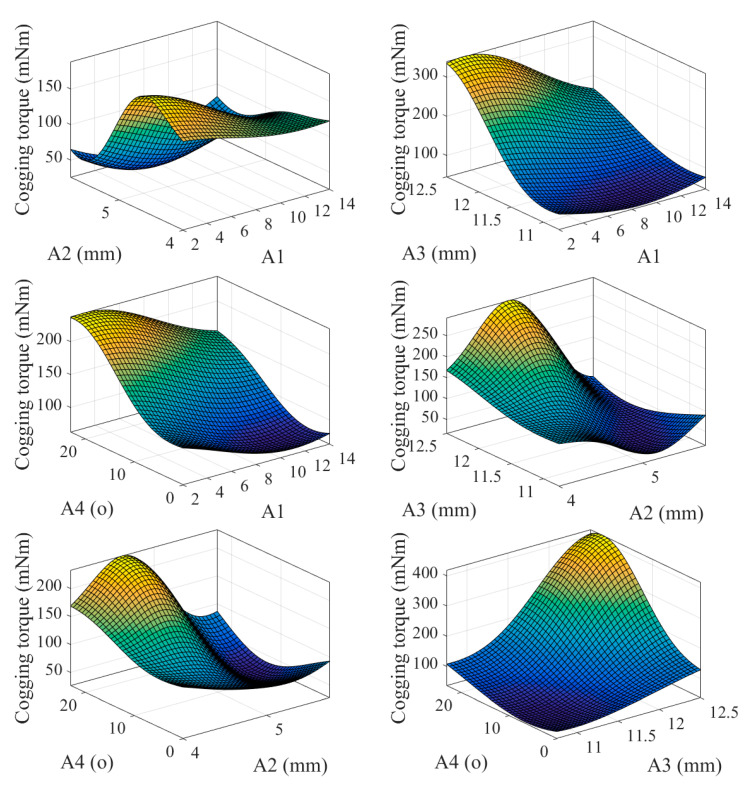
The response surface results of the relationship between the two factors.

The simulation of cogging torque was analyzed. Cogging torque has a bad effect on the motor, because it will cause the motor to generate ripple, vibration, and noise. Therefore, the peak cogging torque is the basis for affecting the difficulty of starting the motor. In this study, uniform design experiment method (UDE) was used to optimize the FSPM structure to reduce cogging torque. The factor codes of UDE are shown in Table 5. Table 6 shows the results of orthogonal arrays and various levels of corresponding cogging torque. The orthogonal arrays were composed of four factors and eight sets of various levels of parameters, and the parameters were substituted into ANSYS Maxwell to obtain the results of cogging torque. Figure 8 shows the response surface chart of the nonlinear relationship between factors A1, A2, A3, A4, and cogging torque. The motor with 5 pole numbers, 5.25 mm rotor-tooth lengths, 11.25 mm magnet length, and 16° rotor-tooth angle shows the lowest cogging torque of 32.7 mNm.

### 3.3. The Manufacturing and Measurement of the FSPM Motor

**Figure 9:**
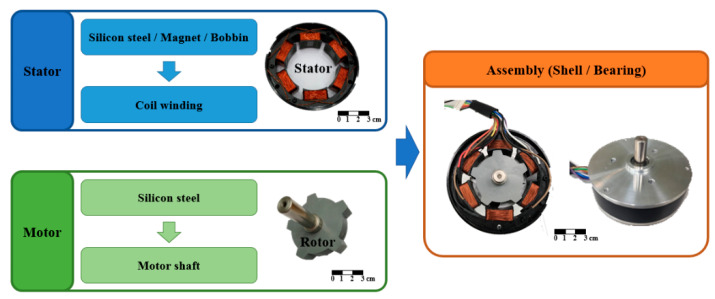
The assembly process of FSPM.

**Figure 10:**
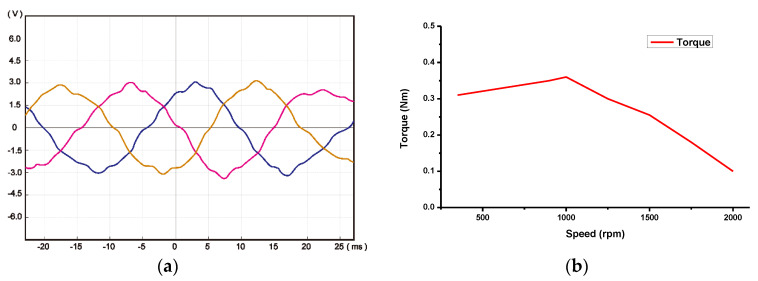
The measurement of (**a**) back-EMF, and (**b**) the N-T curve of FSPM under an input current of 9 A and a rated speed of 1000 rpm.

According to the parameters designed from the previous chapter, the 6/5 FSPM was manufactured with the stator outer diameter as 86 mm, and the effective shaft length as 10.5 mm. The assembly process is shown in Figure 9. The stator part contained the stator silicon steel sheet, permanent magnet, and winding turns. The rotor part only contained silicon steel sheets. The load torque under the different speeds was measured by using the torque sensor and the active motor, and plotted to the N-T curve. Figure 10 shows that the peak value of back-EMF was about 3 V with no load state. The torque output is 0.36 Nm under an input current of 9 A and a rated speed of 1000 rpm.

### 3.4. Experimental Results of Model Predictive Current Control

**Figure 11:**
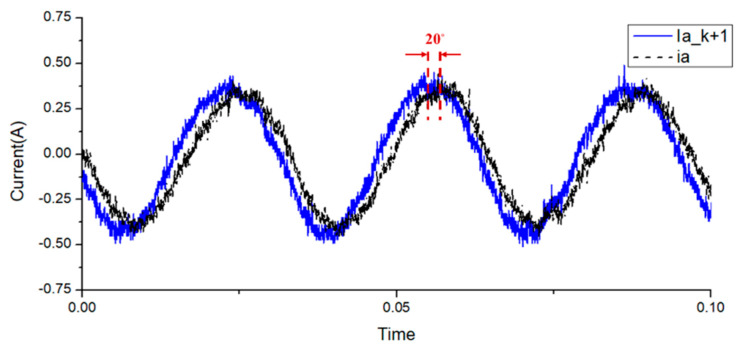
Modified MPCC current prediction analysis, where the solid line is the predicted A-phase current, and the dotted line is the measured A-phase current.

**Figure 12:**
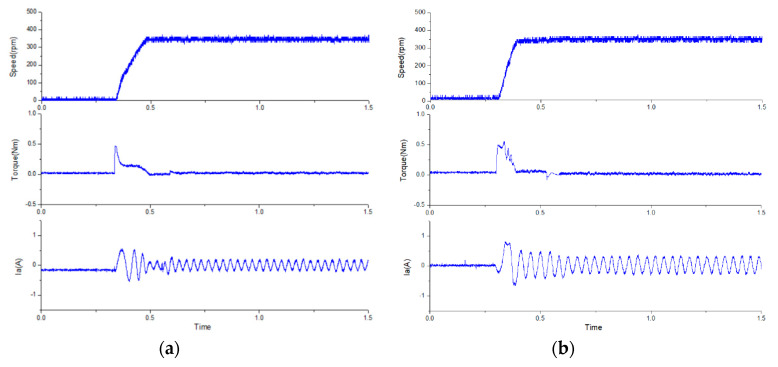
The step response for no load at a speed of 350 rpm: (**a**) the traditional PI control, and (**b**) the proposed modified MPCC.

To compare the dynamic and steady-state performances of the proposed improved MPCC, a traditional PI control architecture (with SVM) [32] was used for comparison. The control parameters for the current loops of the traditional PI control architecture and the improved MPCC were the same, in which the phase margin was set as ~60°, *K_p_* was set as 3, and *K_i_* was set as 3500. For the speed loops, the controller gain of traditional PI control architecture was also set as ~60°, *K_p_* was set as 0.2, and *K_i_* was set as 41. However, the improved MPCC without controller gain was directly adjusted by the modelling. First, the performance of the proposed modified MPCC method was analyzed, as shown in Figure 11. Figure 11 shows the relationship between the measured A-phase current and predicted A-phase current. It can be seen that the predicted A-phase current had a lead angle of about 20 degrees, compared to the fed back A-phase current. This shows the fast response characteristics of the current prediction model. The step responses of the two methods at no load were compared, as shown in Figure 12, from rest to 350 rpm. From the top to bottom, the waveforms are the speed, torque, and A-phase current. It can be found that in the proposed modified MPCC, the slope of the speed is closer to linear, and the improvement effect can be clearly observed from the trend of the torque. The reason is that the proposed improved MPCC is more complete for motor dynamics considerations, which could improve the non-linear torque of the FSPM and achieve better dynamic performance. In addition, the traditional PI control is also affected by the non-linear torque, which makes the required acceleration time longer.

**Figure 13:**
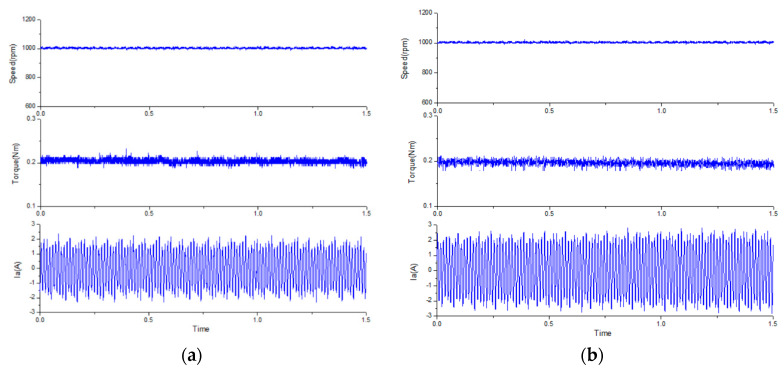
The steady-state response for 0.2 Nm load at a rated speed of 1000 rpm: (**a**) the traditional PI control, and (**b**) the proposed modified MPCC.

**Figure 14:**
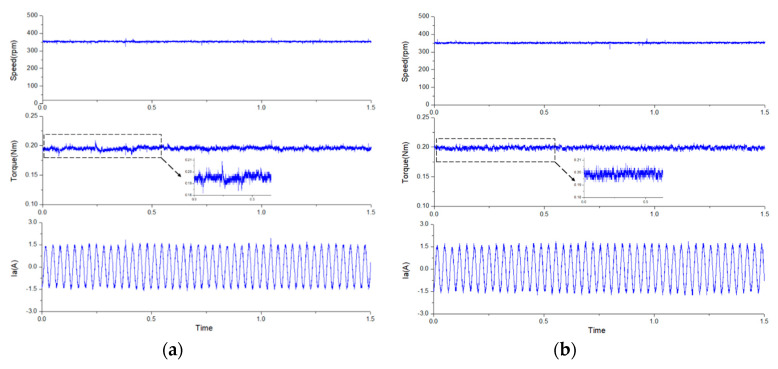
The steady-state response for 0.2 Nm load at a speed of 350 rpm: (**a**) the traditional PI control, and (**b**) the proposed modified MPCC.

Figure 13 shows the steady-state performance of the two methods with 0.2 Nm load at 1000 rpm. From the top to bottom, the curves are rotor speed, electromagnetic torque, and one-phase stator current. The ripple of the torque and speed of the two methods are also very similar at rated speed. However, when the speed drops to 350 rpm, the difference between the two methods begin to differ significantly, as shown in Figure 14. It can be found that the proposed modified MPCC had a torque ripple of 5.6%, which has a lower torque ripple than the conventional PI control (10%). According to [26,33], it can be known that excessive torque ripple causes unstable speed and lower efficiency. Therefore, it is ideal to maintain the torque ripple below 10% in general.

**Figure 15:**
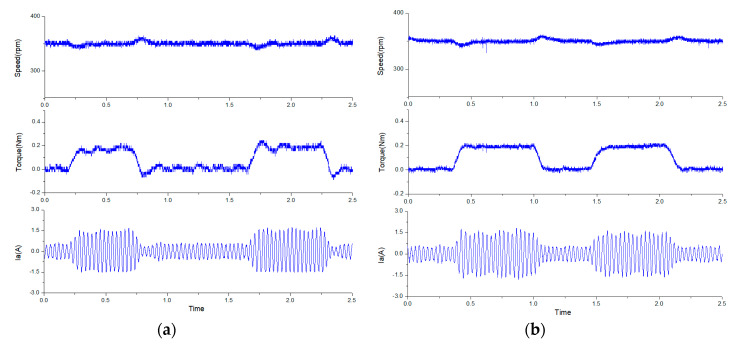
The dynamic response for disturbance load of 0.2 Nm at a speed of 350 rpm: (**a**) the traditional PI control, and (**b**) the proposed modified MPCC.

In addition, an external load disturbance was tested to verify that the proposed modified MPCC could achieve better dynamic performance than the traditional PI control. The dynamic test for a disturbance load of 0.2 Nm is shown in Figure 15, at a speed of 350 rpm. When a disturbance of rated half-load was applied to the FSPM, the speed dropped slightly and then ran quickly to its original reference speed. It can be found that the proposed modified MPCC had a better dynamic performance than the traditional PI control, and lower torque ripple.

**Figure 16:**
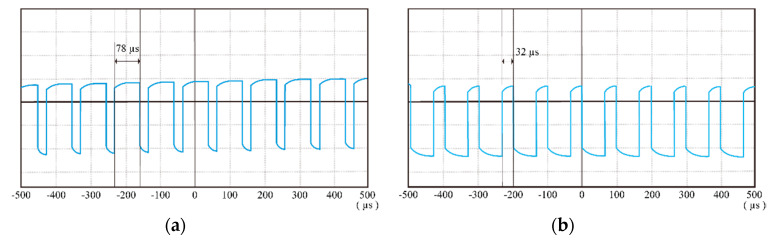
The execution times of: (**a**) traditional method, and (**b**) improved MPCC method.

Therefore, the proposed modified MPCC effectively improved torque ripple for better steady-state performance at the low speed. In the follow-up, the dynamic characteristics of the FSPM at the low speed are discussed in detail. The execution times in both implementations are shown below. The execution times of the traditional method and the improved MPCC method were 78 μs and 32 μs, respectively, as shown in Figure 16. The results show the improved MPCC method with shorter execution time. The MCU was able to get more working bandwidth.

## 4. Conclusions

This paper presents the design of an FSPM and an improved control system to enhance FSPM performance and torque sensing investigation. In the motor design, the FSPM of six stators slots with five rotor poles was investigated by experimental design. The analytical magnetic circuit design was used to determine the related motor parameter. The results show that the lengths of magnet and airgap were defined as 6.9 mm and 0.5 mm, respectively. The cross-sectional area was the magnetic flux passing area. The B_m_ and B_g_ were calculated as 0.88 T and 0.85 T, respectively. The simulation result of B_g_ was about 0.8–1.0 T, which was similar to the calculation result of 0.85 T. The torque output was 0.36 Nm under an input current of 9 A and the rated speed of 1000 rpm. Furthermore, the results show that the lowest cogging torque was 32.7 mNm. In the control of FSPM, a modified MPCC strategy was able to present a better performance response and decrease the computational burden. In step response tests of 350 rpm, the proposed modified MPCC achieved better dynamics. In addition, the nonlinear problem of the startup speed was effectively solved. The torque sensing at different speeds was obtained. In the steady-state performance with 0.2 Nm load at 1000 rpm, the ripple of the torque and speed of the two methods were similar. However, when the speed dropped to 350 rpm, torque ripple was improved from 10% to 5.6%. In the dynamic test for a disturbance load, it was found that the proposed modified MPCC had a fast dynamic performance and lower torque ripple. Therefore, the proposed modified MPCC effectively improved torque ripple for better steady-state performance at the lower speed.

## Figures and Tables

**Table 1:** The specification of the as-designed 6/5 FSPM.

Specification	Unit	Value
Rated power	W	40
Rated speed	rpm	1000
Rated voltage	V	3
Stack length (L)	mm	10.5
Magnet	--	NdFeB N30
Steel Sheets	--	35CS440

**Table 2:** The winding figuration of the 6/5 FSPM.

Phase	A		B		C	
**In**	1	4	2	5	3	6
**Out**	2	5	3	6	4	1

**Table 3:** The parameters of the as-designed 6/5 FSPM.

Parameters	Unit	Value
*w_t_, w_o_, w_y_, l_m_, w_r_*	mm	6.9
*R_so_*	mm	43
*R_si_*	mm	26.5
*R_ro_*	mm	26
*l_a_*	mm	11.25

**Table 4:** The parameters of the designed FSPM.

Parameters	Unit	Magnet	Air Gap
Length	mm	6.9 (*l_m_*)	0.5 (*l_g_*)
Cross-sectional Area	mm^2^	112.88 (*A_g_*)	72.03 (*A_m_*)
Relative Permeability	--	1.05 ($\mu_{r}$)	1 ($\mu_{0}$)
Flux Density	Tesla	0.88 ($B_{m}$)	0.85 ($B_{g}$)

**Table 5:** The factor codes of $U_{8}^{*}\left(8^{5}\right)$

...	Factor	Unit
A1	Pole numbers	-
A2	Rotor-tooth lengths	mm
A3	Magnet length	mm
A4	Rotor-tooth angle	°

**Table 6:** The orthogonal arrays of $U_{8}^{*}\left(8^{5}\right)$.

	A1	A2	A3	A4	Cogging Torque (mNm)
**1**	2	4.25	11.5	23	204.4
**2**	4	4.75	12.5	20	518.9
**3**	5	5.25	11.25	16	32.7
**4**	7	5.75	12.25	13	40.3
**5**	8	4	11	10	113.7
**6**	10	4.5	12	6	114.1
**7**	11	5	10.75	3	34.2
**8**	14	5.5	11.75	0	78.7
